# Chemically Induced Compatible Interface in Pyrolyzed Bacterial Cellulose/Graphene Sandwich for Electrochemical Energy Storage

**DOI:** 10.3390/ma15196709

**Published:** 2022-09-27

**Authors:** Xiangjun Wang, Zhichang Xiao, Xinghao Zhang, Debin Kong, Bin Wang, Peng Wu, Yan Song, Linjie Zhi

**Affiliations:** 1 School of Chemical and Biological Engineering, Taiyuan University of Science and Technology, Taiyuan 030021, China; 2 Department of Chemistry, College of Science, Agricultural University of Hebei, Baoding 071001, China; 3 School of Materials Science and Engineering, China University of Petroleum, Qingdao 266580, China; 4 CAS Key Laboratory of Nanosystem and Hierarchical Fabrication, CAS Center for Excellence in Nanoscience, National Center for Nanoscience and Technology, Beijing 100190, China; 5 Computer Engineering Department, Taiyuan Institute of Technology, Taiyuan 030008, China; 6 Key Laboratory of Carbon Materials, Institute of Coal Chemistry, Chinese Academy of Sciences, Taiyuan 030001, China

**Keywords:** chemical bonding interface, mass transfer efficiency, electrode material

## Abstract

Herein, a three-step approach toward a multi-layered porous PBC/graphene sandwich has been developed, in which the chemical bonding interactions have been successfully enhanced via esterification between the layers of pyrolyzed bacterial cellulose (PBC) and graphene. Such a chemically induced compatible interface has been demonstrated to contribute significantly to the mass transfer efficiency when the PBC/graphene sandwich is deployed as electrode material for both supercapacitors and lithium–sulfur batteries. The high specific capacitance of the supercapacitors has been increased by three times, to 393 F g^−1^ at 0.1 A g^−1^. A high initial discharge specific capacity (~1100 mAhg^−1^) and high coulombic efficiency (99% after 300 cycles) of the rPG/S-based lithium–sulfur batteries have been achieved.

## 1. Introduction

The increasingly serious problem of energy shortage and environmental pollution urgently requires a reduction in the world’s dependence on fossil energy sources [1,2,3]. Supercapacitors consisting of two electrodes separated by a separator, are promising energy storage devices owing to their long cycle life, high safety, and short charging time [4,5,6]. It has been proven that bacterial cellulose (BC) holds great promise as either the precursor or backbone of supercapacitor electrode material [7,8,9], due to its porous 3D network. Composites of BC with conductive polymers and carbon materials were synthesized and applied as electrode materials for supercapacitors and batteries [10,11,12]. However, the low specific surface area (SSA) limits their capacitance considering the insufficient electronic contact sites [13,14]. To increase the SSA and the mechanical strength of BC, graphene with an ultrahigh theoretical SSA of graphene (2630 m^2^ g^−1^) has been employed for BC/graphene composite in lithium–sulfur batteries [15]. It was reported that the hybrid materials composed of two-dimensional graphene and one-dimensional nanomaterials have the advantages of surface area [16] and electrical conductivity [17,18]. Graphene-based derivatives with nature-derived biodegradable organic supercapacitor electrodes have been reported [19,20]. Excellent recyclability was reported for BC/graphene/polypyrrole composites as working electrodes for supercapacitors, which is mainly contributed by the 3D interpenetrating network formed from the cooperation of BC and graphene [21]. The free-standing conductive polymer electrodes on high-performance flexible supercapacitors were developed with BC and graphene as the backbone [22].

To increase the electron conductivity, pyrolysis was employed for biomass while the microstructure could be largely maintained. The activated carbon originated from bamboo, and maintained the fibrous microstructure of the bamboo, which was employed as the carbon matrix of gold nanoparticles [23]. Due to the unique structure with highly mineralized chitin–protein fibers, crab shells can be used as bio-templates to fabricate hollow carbon nanofibers; these fibers can then be the host of the other material, either to encapsulate sulfur or silicon to form electrodes for Li-ion batteries. The hollow nanostructures of pyrolyzed crab shells provide sufficient space for the volume expansion of sulfur silicon during the discharge/charge processes [24]. Analogously, pyrolysis has been widely employed to prepare pyrolyzed bacterial cellulose (PBC) as the electrode material in supercapacitors [10,11,25,26,27], which resulted in a nanoribbon-shaped 3D carbon network, with abundant porosity originating from BC [28,29,30,31]. Hence, the composites of PBC/graphene instead of BC/graphene have the advantage of higher conductivity. The PBC/graphene composite was simply prepared by physical mixing and employed as an interlayer for high-performance lithium–sulfur (Li–S) batteries [15]. A more complicated method has been applied to prepare PBC/graphene composite via membrane–liquid interface culture followed by freeze-drying and canonization, which provides good electrochemical performance in supercapacitors, even upon a bending–releasing test for flexible devices [32].

However, the interfaces either between BC and graphene or between PBC and graphene are commonly inefficient. On one hand, the limitation of the solubility of BC in an aqueous solution lowers the chemical reaction sites between BC and graphene. On the other hand, the pyrolysis process after the fabrication of BC/graphene composite destroys the possible chemical connection between BC and graphene, and the 3D network could collapse due to the shrinkage of the bacterial cellulose during the pyrolysis [33,34,35]. Consequently, both the electron transfer rate inside the materials and the ion transfer rate are retarded. Most of the interfaces between PBC and graphene are physical combinations without chemical bonding, which would lead to a serious blockage of electron transfer and ion transfer. Therefore, developing an efficient synthetic methodology to induce a chemically compatible interface between PBC and graphene is highly required but remains a great challenge.

**Figure 1:**
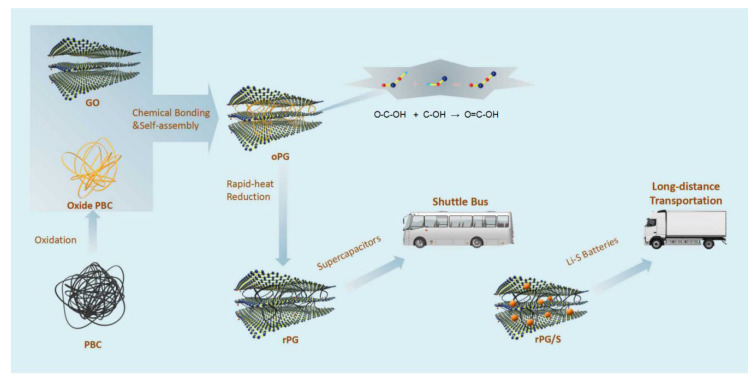
Illustration of rPG-based electrode configuration and representative application in supercapacitors and lithium–sulfur batteries.

Herein, a facile pre-oxidation of PBC is introduced in this paper, followed by an engineered self-assembly method, and an ultra-rapid thermal reduction process, where graphene oxide (GO) is bridged by oxidized PBC hinges through chemical bonding interactions, thus forming PBC oxide (oPG)-impregnated assemblies of a multi-layered oriented GO sandwich with a compatible composting interface. After an elaborate and ultra-rapid reduction, a multi-layered reduced PBC/graphene (rPG) sandwich with high surface area, robust bi-continuous network, superficial oxygen-containing groups, and exceptional electrochemical properties can be obtained. These features not only provide a continuous conductive network but also facilitate the easy access of electrolytes, and enables superior mass transfer. Remarkably, compared to reduced PBC (rP), rPG exhibits a three-times-higher specific capacity (393 F g^−1^) at a current density of 0.1 A g^−1^ in supercapacitors. In addition, considering its abundant porosity, rPG has also been utilized for sulfur storage as the cathode of Li-S batteries. Hence, rPG not only enhances the conductivity of the cathode but also limits the sulfur in the cathode by designing the porous structure as a physical barrier. Electrochemical analysis showed that the rPG/sulfur composite possesses excellent cycling stability (a decay of 0.09% per cycle after 300 cycles) and coulomb efficiency in Li-S batteries and a significantly improved rate performance in both high-power supercapacitors and high-energy Li-S batteries (Figure 1). Therefore, this work provides an effective strategy to develop advanced porous carbon materials for practical applications both in supercapacitors and Li-S batteries.

## 2. Materials and Methods

### 2.1. Materials

Hainan Nanye Industry Company kindly supplied the bacterial cellulose pellicles. The obtained BC pellicles were prepared by freeze-drying (−54 °C, 48 h). The obtained BC aerogel was then pyrolyzed under flowing argon to obtain pyrolyzed BC (PBC). Then, the PBC was treated with nitric acid to obtain the PBC oxide. The rP was obtained through the reduction of PBC oxide at 900 °C for 10 s. The self-assembly of PBC oxide and GO was at room temperature, and hydrochloric acid was slowly added to cause the GO sheets to overplay together, which formed the sandwich structure. With the same rapid thermal processing as rP, the reduced sandwich composite, rPG, was obtained.

### 2.2. Characterization of Materials

The Hitachi S-4800 instrument was employed to obtain the scanning electron microscopy (SEM) micrographs. The powder X-ray diffraction (XRD) measurements were carried out on a Rigaku D/max 2500 instrument with Cu Kα irradiation. The N2 adsorption isotherms were obtained with a Micromeritics accelerated surface area porosimetry (ASAP 2020, Jackson, MS, USA) auto adsorption analyzer at 77 K, and the SSA was obtained through Brunauer–Emmett–Teller (BET) analyses of the adsorption isotherms.

### 2.3. Electrochemical Performance

The electrochemical comparison of rP and rPG as the active materials of the supercapacitor electrode was carried out by the two-electrode symmetric supercapacitor systems. The electrode materials were made by mixing the active materials (rP or rPG), the polytetrafluoroethylene (PTFE) binder, and acetylene black with a weight ratio of 8:1:1. The active materials and the acetylene black were mixed in an agate mortar until a homogeneous black powder was achieved. PTFE was then added to the mixture with a few drops of water. The obtained paste was pressed to a piece of round-shaped nickel foam at 10 MPa with a diameter of 1.0 cm, and then dried in a vacuum oven at 100 °C for 12 h. The weight of the active materials on each electrode was 1 mg. The coin-type cells were assembled with the electrode materials, the separator made of glass fiber, and the electrolyte of 6 M KOH aqueous solutions. The CHI660D electrochemical station (CH Instrument, Shanghai, China) was employed to obtain the electrochemical properties of the cells. The EIS plots were tested in the frequency range from 100 kHz to 0.1 Hz at 1 V scanning amplitude. The CT2001A Battery Program Controlling Test System (China-Land Com. Ltd., Wuhan, China) was employed to take the galvanostatic charge/discharge measurements.

The cathode electrode for Li-S batteries was different from that for supercapacitors. It was made by mixing rPG/S (or rP/S composites), acetylene black, and poly (vinylidene fluoride) (PVDF) with the mass ratio of 8:1:1. PVDF was used as the binder in N-methylpyrrolidone (NMP) solvent dispersant to get uniform slurry. Instead of pressing on the nickel foam, the cathode electrode for Li-S battery was spread onto an aluminum foil with a blade. In order to avoiding contact with oxygen, the Li-S batteries were assembled in an Ar-filled glove box, and the coin cells were CR2032. The separator was a Celgard 2400 membrane and Li was the anode with 1 mol L^−1^ lithium bis(trifluoromethanesulfonyl) imide and 2 wt.% LiNO_3_ dissolved in tetraethylene glycol dimethyl ether (TEGDME). According to the calculation, the areal loading of sulfur for each coin cell was ~2.5 mg cm^−2^ within a voltage range from 1.7 V to 2.8 V. The galvanostatic tests, cycle tests, and rate tests were carried out with the Land Battery Measurement System, where 1 C (1675 mA g^−1^) was equal to a full discharge or charge in an hour. All the specific capacity was calculated based on the mass of sulfur. CV measurement was conducted on a Bio-logic VMP potentiostat at a scanning rate of 0.05 mV s^−1^ in the voltage range between 1.7 V and 2.8 V.

## 3. Results and Discussion

### 3.1. Characterization of PBC, rP and rPG

**Figure 2:**
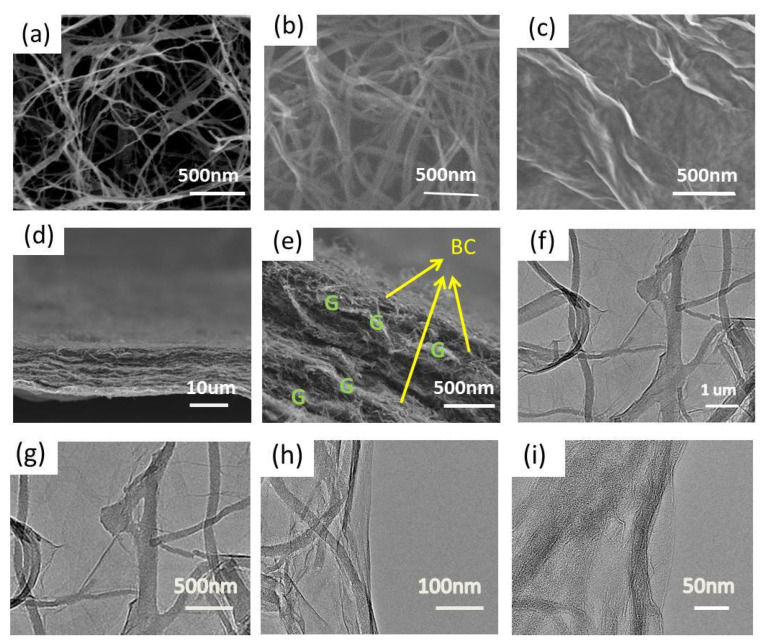
Microstructure characterization. SEM images of (**a**) PBC, (**b**) rP, (**c**) GO, (**d**,**e**) cross section of rPG, (**f**–**i**) TEM image of rPG with different magnifications.

The PBC was synthesized by freeze-drying bacterial cellulose and through post-pyrolysis as reported before [30]. During the composite process between PBC and graphene, chemical oxidation and reduction were deployed successively to obtain the homogeneous composite. To investigate the contribution of GO and the superiority of the sandwich structure, a control sample of rP without graphene oxide was also fabricated. As depicted in Figure 1, the oxidation of PBC was acheived to introduce oxygen groups, so that oPG and GO would be composed through the self-assembly due to the functional groups’ interaction between GO and oPG. To increase the electric conductivity, an ultra-rapid thermal reduction process within 10 s at 900 °C was employed to obtain rPG [36]. A similar method was used to prepare rP without GO. The three-dimensional porous architectures of BC networks consist of straight nanoribbons (Figure S1a) that can be maintained after pyrolysis (Figure 2a). However, the oxidation by HNO_3_ destroys the original cross-linking configurations among the nanoribbons of the PBC. In contrast, the compatibility of PBC oxide with GO is increased, and the three-dimensional sandwich structure is rebuilt when oxide PBC is embedded within GO backbones (Figure 2d and Figure S1b). The rPG sustains the original GO wrinkles and assembly layer by layer (Figure 2e and Figure S1c). Furthermore, the transmission electron microscope (TEM) image confirms the existence of macrospores within rP (Figure S1d), while that of rPG shows an obvious new class of corrugations with tens of micrometers in width (Figure 2e–h). Accordingly, graphene sheets are successfully introduced to PBC by self-assembly and ultra-rapid thermal reduction.

**Figure 3:**
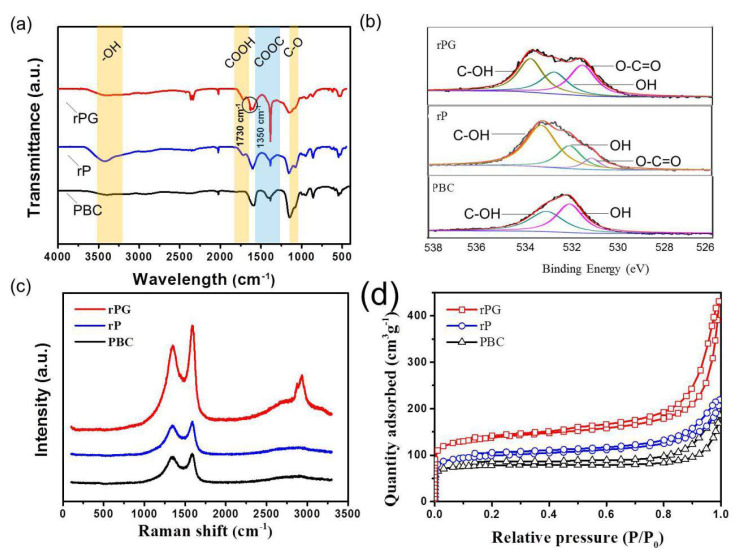
Composition and pore structure characterization. (**a**) FTIR, (**b**) XPS high-resolution spectra of O1s, (**c**) Raman and (**d**) N2 adsorption and desorption curve of PBC, rP and rPG.

The chemical nature of the functionalities was examined using FTIR spectroscopy as shown in Figure 3a. The typical peak at ~1585 (black circled) for rPG can be assigned to the stretching vibration of the quinoid and benzenoid structure of graphene [37], and rPG has a C-O stretching vibration at 1087 cm^−1^ [38]. The spectra of PBC, rP, and rPG display the main characteristic bands at 3345 cm^−1^ (stretching vibration of OH groups), and 1050 cm^−1^ (stretching vibration of C–O bonds) [39]. New peaks at 1730 cm^−1^ appear in the spectra of rP and rPG, originating from the introduction of carboxyl groups through the oxidation of PBC and the addition of GO [40], which are partially preserved during the ultra-rapid thermal process. Additionally, a sharp new peak at ~1350 cm^−1^ appeared with the addition of GO originating from the esterification between OH groups and carboxyl groups, which corroborates the successful chemical bonding inside the target rPG composite. X-ray photoelectron spectroscopy (XPS) was further adopted to investigate the surface chemistry of rPG and control samples. Table S1 shows the element content changes of PBC, rP, and rPG. After oxidation and reduction, rP had higher oxygen content with lower carbon content compared with PBC. The carbon content of rPG was similar to that of rP. Both rP and rPG exhibited primary graphitic C1s peaks at 285 eV and an O1s peak at 532 eV as shown in Figure S2a. The C1s spectra of PBC, rP, and rPG show approximately regular changes (Figure S2b). The deconvolution of the C1s spectrum of PBC yielded two peaks. The main peak at 284.8 eV is associated with sp^2^-C in the PBC nanofibrils considering the presence of graphitic layers [41]. The relatively weak peak at 285.6 eV is assigned to carbon atoms in C=O, validating the existence of a small portion of oxygen-containing functionalities in PBC nanofibrils. Along with the oxidation process and addition of GO, the original sp^2^-C characteristic peaks at 284.8 eV were retained for rP and rPG. However, the C=O peak redshifted to 286.3 eV for rPG, and the new peaks at 289.2 eV for both rP and rPG belonging to O–C=O [40] were introduced during the oxidation reaction. As shown in Figure 3b, the deconvolution of O1s indicated the difference in the oxygen functional groups among PBC, rP and rPG. Two peaks were identified in the O1s core-level spectra of PBC at 533.03 eV and 532.03 (C=O), which belong to the COOH group [42]. The sharp increase in the intensity of the peak at 533.03 eV of rP revealed that the ether bond was preferentially introduced upon the oxidation, compared with that of PBC, which remained partially after composing with GO and the mild reduction process. Moreover, an additional peak (531.78 eV) was identified in the O1s core-level spectra of rP that corresponded to the carbonyl group. The distribution of oxygen groups in the rPG was comparable, while the relative intensity of the carboxyl group peak was even much higher in the case of rPG. This is indicative of the successful compounding process with the higher ratio of the carboxyl group compared with rP due to GO. These results are in good accordance with the FTIR detection.

The graphitic structure of the PBC, rP, and rPG is characterized by Raman spectra (Figure 3c). The intensity ratios between the D band at 1361 cm^−1^ and the G-band at 1600 cm^−1^ (ID/IG) of PBC, rP, and rPG are 0.92, 0.93, and 0.87, respectively. The ratio of ID/IG was lowered for rPG compared with PBC and rP, indicating the decrease in disorder due to the introduction of GO. Moreover, the resulting rPG shows an obvious 2D band peak due to the substantial recovery of the basal structure of sp^2^ graphitic domains in the GO samples. Figure S3 shows the characterization of the rP and rPG by means of X-ray diffraction (XRD). The peaks of rPG around 22.5 and 43.8°, assigned to the (002) and (100) planes, seem the same as those of rP with almost invisible intensity due to the similar and compatible crystalline structure between rP and GO. All of these structural characteristics render rPG a promising candidate as an electrochemical electrode material.

Figure 3d depicts a BET nitrogen adsorption–desorption isotherm of PBC, rP, and rPG at 77 K. The Brunauer–Emmett–Teller (BET) analysis disclosed that the SSAs for PBC, rP, and rPG were 336.5 m^2^ g^−1^, 264.7 m^2^ g^−1^ and 469.9 m^2^ g^−1^, respectively (Table S2). The prominently decreased SSA of rP in comparison with that of PBC is due to the rearrangement of the nanoribbons during the oxidation. Table S1 suggests that the surface area of the micropores of rPG (315.4 m^2^ g^−1^) increased compared with that of PBC (242.8 m^2^ g^−1^) and rP (227.8 m^2^ g^−1^), which enables more adsorption sites of the electrodes, resulting in a higher capacity. Meanwhile, the ratio of the surface area of the micropores and the BET surface area decreased, indicating that rPG has relatively more mesopores and macropores, which can enhance the mass transfer efficiency, leading to the good rate ability at a higher current density. The pore distributions (Figure S4) show that the micropore size of the PBC was mainly between 0.7 and 1.8 nm, and similar distributions could also be observed for the rP samples. In comparison, the micropore sizes of the rPG samples significantly increased in a range from 0.6 to 0.7 nm, which is beneficial for improving the charge storage ability as the electrode material for supercapacitors [42]. Furthermore, the appearance of mesopores between 10 and 40 nm grants the possibility of mass transition inside the electrode materials. Consequently, we envisage that rPG can also be utilized as the carbonaceous matrix for elemental sulfur, and the sulfur-loading content in the rPG/S composite was calculated to be 63.3 wt.% using thermogravimetric analysis (TGA) (Figure S5). For comparison, sulfur with equivalent content was also loaded into the PBC host to obtain the PBC/S composite.

### 3.2. Electrochemical Characteristics of rP and rPG

#### 3.2.1. Supercapacitors

**Figure 4:**
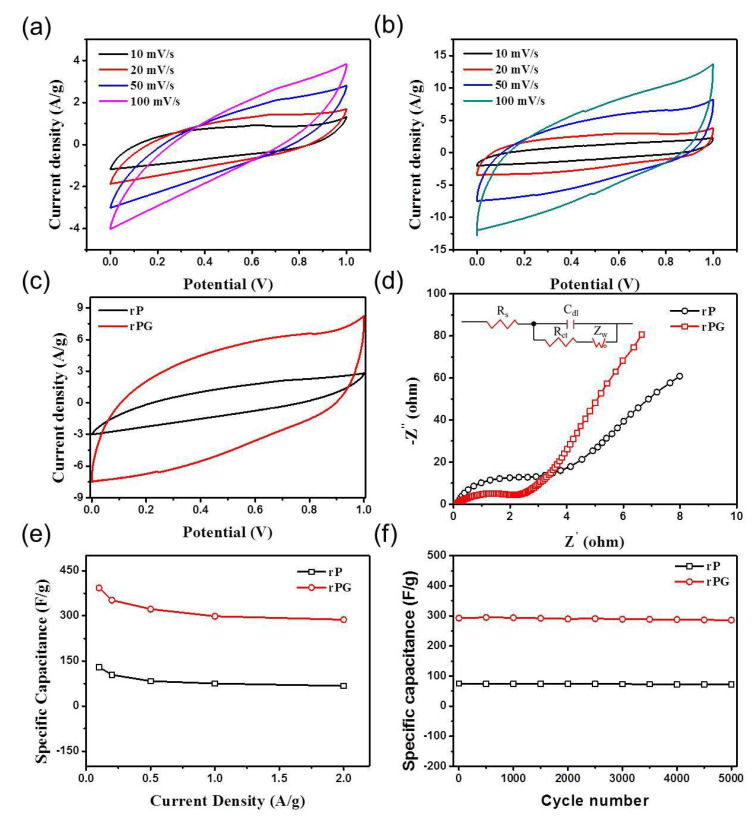
Electrochemical performance of rP and rPG. CV curves of (**a**) rP and (**b**) rPG at scan rates of 10, 20, 50, and 100 mV/s over a potential range from 0 to 1 V. (**c**) comparative CV curves of rP and rPG at scan rates of 50 mV/s over a potential range from 0 to 1 V. (**d**) Nyquist plots of rP and rPG in the frequency range from 100 kHz to 0.1 Hz (inset is the corresponding plots of high-frequency ranges). (**e**) Specific capacitances at various current densities. (**f**) cycling performance during 5000 cycles.

The electrochemical performances of rP and rPG in supercapacitors were investigated with a 6 M KOH aqueous electrolyte in coin cells. Figure 4a and b show cyclic voltammogram (CV) curves of rP and rPG, respectively. Both of the samples exhibited a typical capacitive behavior with rectangular-shape voltammetry characteristics from 0 to 1 V over a wide range of voltage scan rates. However, the obvious differences between the CV curves of the two samples highlight both the shapes and the integral areas, especially at the scan rate of 50 mV s^−1^ (Figure 4c). Furthermore, over the triple integral area, the CV curves of the rPG-based supercapacitor exhibit humps as well as a rectangular shape, indicating that the capacitance of rPG increased and the redox reaction was the capacitive response resulting from the combination with the electrical double-layer formation and redox reactions.

In order to understand the electron transport properties in supercapacitors, we further measured the electrochemical impedance spectroscopy (EIS) in a frequency range of 0.1 Hz–100 kHz. The Nyquist plots are shown in Figure 4d. The first intersection of the curve with the real axis in the Nyquist plots refers to the solution resistance (R_S_) [43]. Both of the intersections of rP and rPG are no more than 0.5 Ω, indicating that there are accessible ion transmission channels inside the two electrode materials [44]. Generally, the semicircle at a high frequency (in the kHz range) is attributed to the charge transfer resistance (R_ct_) at the electrode/electrolyte interface, associated with the surface properties of the porous electrode and corresponding to the faradic charge transfer resistance. Obviously, the R_ct_ values of rPG are much lower than that of the rP samples, confirming a much smoother mass transfer process and the advantage of compatible interfacial structure between PBC and graphene. In this regard, three increments of the specific capacitances (i.e., the capacitances of rP and rPG are 129 F g^−1^ and 393 F g^−1^ at 0.1 A g^−1^, respectively, as shown in Figure 4e) can be observed. Even after 5000 cycles at 2.0 A g^−1^, it still retained 97% retention with an excellent specific capacitance of 286 F g^−1^, which is much higher than that of rP (72.6 F g^−1^) (Figure 4f). The rPG electrode showed obvious advantages in terms of electrochemical properties due to the designed sandwich structure, which offers more compact architectures with abundant pore structures. Additionally, the oxidation of PBC and the addition of GO introduced oxygen-containing functional groups, which offer extra pseudocapacitance. Consequently, the rPG sandwich provides double-layer capacitance as well as pseudocapacitance as the electrode for supercapacitors.

#### 3.2.2. Li-S Batteries

**Figure 5:**
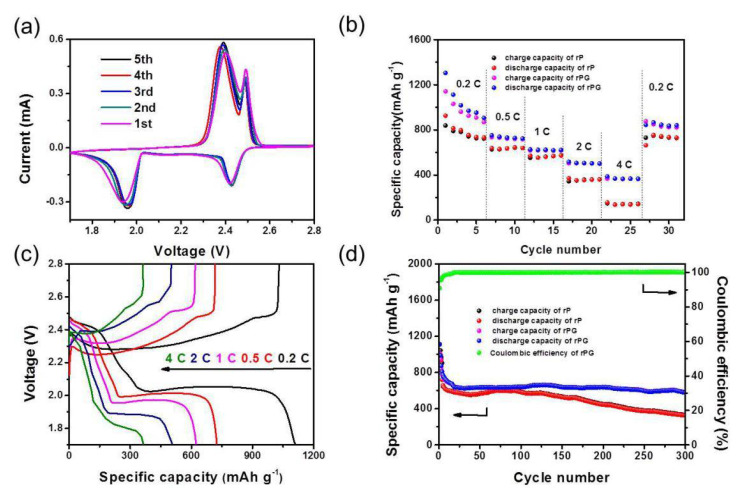
Electrochemical characterization of rPG/S cathode in Li-S batteries. (**a**) cyclic voltammetric (CV) curve between 1.7 V and 2.8 V for the first 5 cycles (scan rate: 0.05 mV s^−1^); (**b**) the rate capability and (**c**) the charge/discharge curves at various current densities from 0.2 to 4 C in the potential range of 1.7–2.8 V; (**d**) The cycling performance at 1 C.

The electrochemical properties of the rPG in Li-S batteries were also evaluated. The CV curve of rPG/S was first tested with CR2032 coin cells between 1.7 V and 2.8 V for the first five cycles (Figure 5a). During the discharge process, rPG/S presented a pair of reduction peaks around 2.4 V and 1.96 V, corresponding to the transformation procedure from S8 to long-chain polysulfides Li_2_S_x_ (x = 4–8) and Li_2_S_4_ to short-chain polysulfides (solid Li_2_S_2_/Li_2_S), respectively. Significantly, the curves in the subsequent five scans were observed in almost the same shape, and the peak positions are extremely close to each other, indicating the excellent cycling stability of the rPG/S cathode in Li-S batteries. Meanwhile, the rPG/S showed excellent rate capability (Figure 5b) at various rates (i.e., 0.2 C, 0.5 C, 1 C, 2 C, 4 C and 0.2 C). Even at 4 C, the composite can still maintain a capacity as high as ~370 mAh g^−1^. When the rate returns back to 0.2 C, the capacity can still recover to ~840 mAh g^−1^. In contrast, rP/S can only deliver ~135 mAh g^−1^ at 4 C and ~730 mAh g^−1^ back to 0.2 C. Figure 5c represents the typical charge/discharge profiles of rPG/S at various current densities from 0.2 to 4 C in the potential range of 1.7–2.8 V, in which the potential hysteresis between discharge and charge profiles for rPG/S cathode is not obvious even at 2 C, indicating the excellent electrochemical kinetics of sulfur encapsulated in the elaborately designed rPG host. The cycling performance of rPG/S and rP/S were also evaluated at 1 C, as shown in Figure 5d. The rPG/S electrode shows an initial discharge specific capacity of ~1100 mAhg^−1^ and maintains 580 mAh g^−1^ when it comes to the 300th cycle (a decay of 0.09% per cycle based on the fourth cycle). In contrast, although the rP/S delivers an equivalent initial discharge specific capacity of ~1100 mAh g^−1^, a severe decline in specific capacity can be observed. After testing for 300 cycles, the coulombic efficiency of rPG/S can still be as high as 99%. Furthermore, the typical charge and discharge profiles of rPG/S within a voltage window ranging from 1.7 to 2.8 V are exhibited in Figure S6, which is in good accordance with the result of the CV curves and cycling performance. The good cycling performance and excellent rate capability can be ascribed to the rational design of the rPG material, which can be further illuminated as follows: (i) the ideal compatibility between the PBC and graphene phase interface endows an excellent mass transfer path; (ii) the three-dimensional sandwiches provide a proper host for polysulfides; (iii) the porous structure of the sandwich improves the cycling stability; (iv) the polar functional groups in the multilayered composites improve the overall performance of the Li-S batteries.

## 4. Conclusions

In this work, we have successfully designed and fabricated a PBC/graphene sandwich structure with significantly enhanced interfacial compatibility via a rationally designed chemical bonding strategy and an ultra-rapid thermal reduction process. Such integrated electrodes (rPG) not only boost a smoother mass transfer process through abundant pore structures and a compatible compositing interface but provide additional oxygen-containing functional groups via the oxidation of PBC and the addition of GO, which can provide extra pseudocapacitance in supercapacitors. Accordingly, a high specific capacitance of 393 F g^−1^ at 0.1 A g^−1^ has been achieved, which is three times larger than that of rP (129 F g^−1^). At the same time, this newly developed sandwich structure can be used in Li-S batteries as well with greatly improved cycling stability and rate capability. The proposed compatible interface induced by chemical bonding interactions may pave the way to a deepened understanding of the relationship between the mass transfer property and the microstructure of electrode materials, in which the independent role of chemistry needs to be deliberated carefully. Moreover, the sandwich structure composite composed of graphene and PBC shows its unique superiority in both electron transport and mass transfer, which indicates that the similar structure of 2D and 1D nanomaterial could be employed in more electrochemical energy storage devices.

## Supplementary Materials

The following are available online at https://www.mdpi.com/article/10.3390/ma15196709/s1, Figure S1: Microstructure characterization. SEM images of (a) BC, (b) rPG, (c) cross section of rPG and TEM image of (d) rP, Figure S2: XPS spectra of rP and rPG, Figure S3: XRD spectra of rP and rPG, Table S1: Element content of PBC, rP and rPG samples, Table S2: Surface area and porosity of PBC, rP and rPG samples. Figure S4: Pore width distribution of PBC, rP, rPG with the pore width (a) from 0.4 nm to 2 nm (b) from 2 nm to 40 nm, Figure S5: TGA of rP/S and rPG/S under nitrogen atmosphere. Figure S6: Charge/discharge profiles of rPG/S.
