# Supplementary material for: Chemically Induced Compatible Interface in Pyrolyzed Bacterial Cellulose/Graphene Sandwich for Electrochemical Energy Storage
Source: Materials (Basel). 2022 Sep 27;15(19):6709. doi: 10.3390/ma15196709 (PMC9571832; doi:10.3390/ma15196709)
Supplement: Supplementary file 1 [file materials-15-06709-s001.zip › materials-1888999-Supplementary.pdf]

materials

MDPI

Communication

Chemically Induced Compatible Interface in Pyrolyzed Bacterial Cellulose/Graphene Sandwich for Electrochemical Energy Storage

Xiangjun Wang [1,*,†], Zhichang Xiao [2,†], Xinghao Zhang [3], Debin Kong [3], Bin Wang [4], Peng Wu [5], Yan Song [6,*] and Linjie Zhi [3,*]

[1] School of Chemical and Biological Engineering, Taiyuan University of Science and Technology, Taiyuan 030021, China
[2] Department of Chemistry, College of Science, Agricultural University of Hebei, Baoding 071001, China
[3] School of Materials Science and Engineering, China University of Petroleum, Qingdao 266580, China
[4] CAS Key Laboratory of Nanosystem and Hierarchical Fabrication, CAS Center for Excellence in Nanoscience, National Center for Nanoscience and Technology, Beijing 100190, China
[5] Computer Engineering Department, Taiyuan Institute of Technology, Taiyuan 030008, China
[6] Key Laboratory of Carbon Materials, Institute of Coal Chemistry, Chinese Academy of Sciences, Taiyuan 030001, China
* Correspondence: wangxiangjun@tyust.edu.cn (X.W.); songyan@sxicc.ac.cn or yansong1026@126.com (Y.S.); zhilj@upc.edu.cn or zhilj@nanoctr.cn (L.Z.)
† These authors contributed equally to this work.

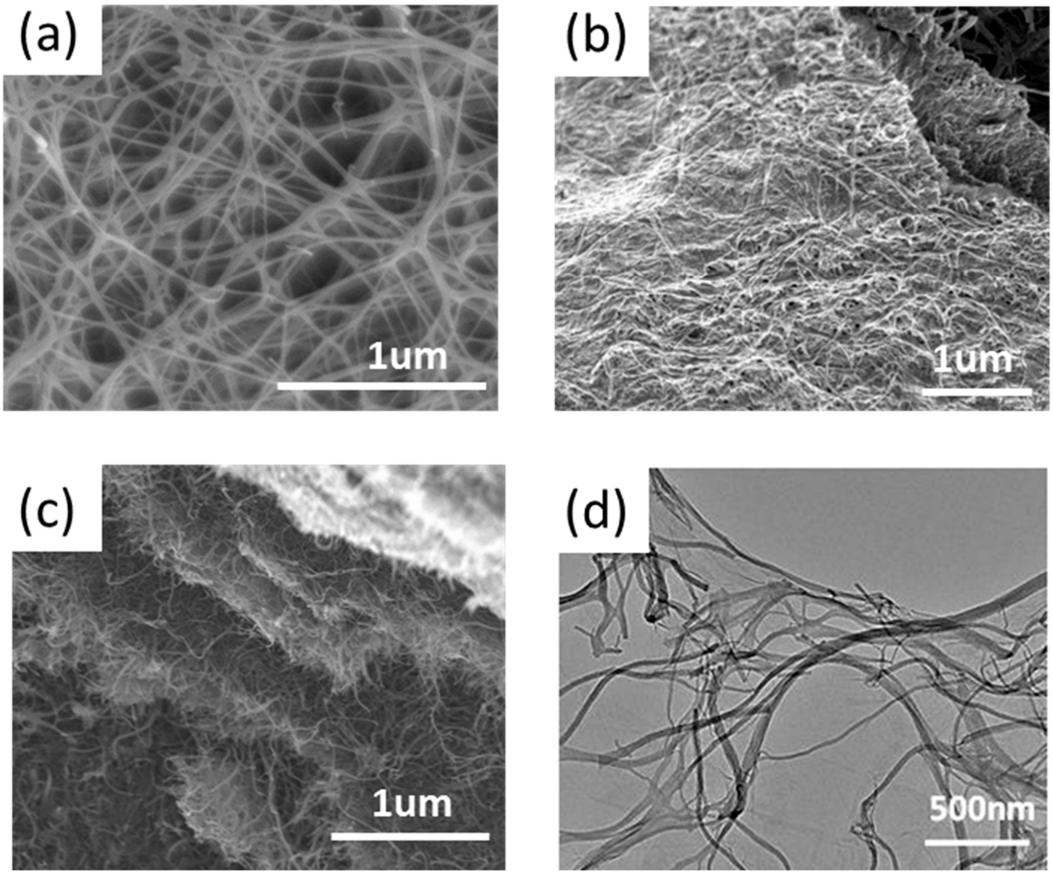

Figure S1. Microstructure characterization. SEM images of (a)BC, (b) rPG, (c) cross section of rPG and TEM image of (d) rP

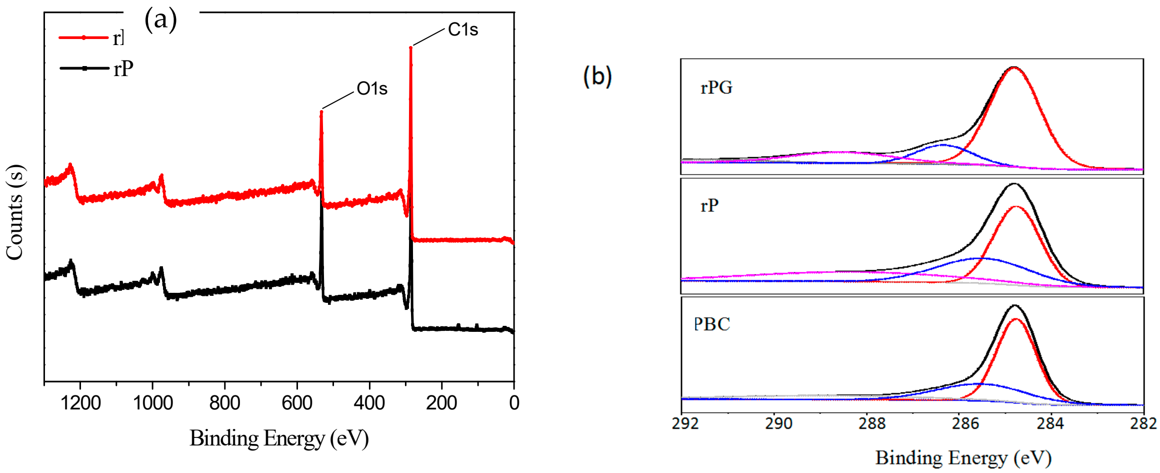

Figure S2. (a)XPS spectra of rP and rPG; (b)XPS high-resolution spectra of C1s of PBC, rP and rPG

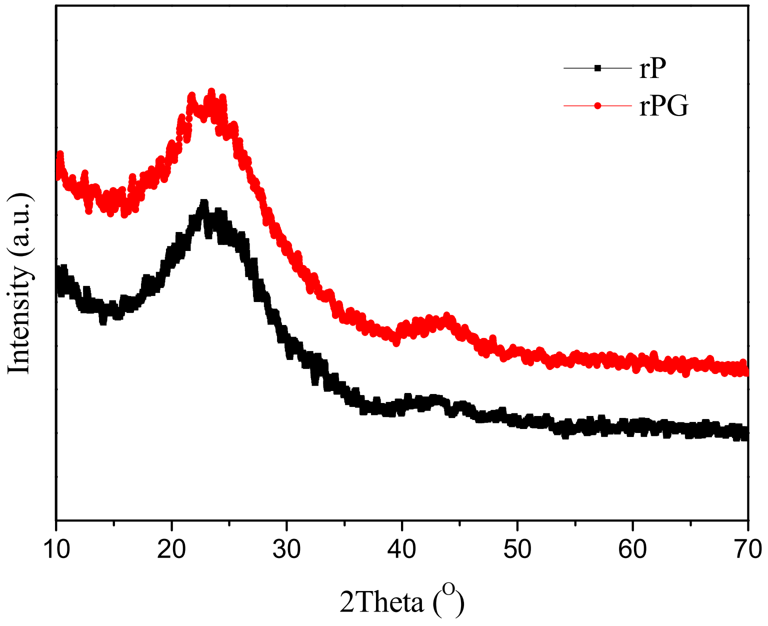

Figure S3. XRD spectra of rP and rPG

Table S1 Element content of PBC, rP and rPG samples

Sample	C	O	Others
**PBC**	90.8	6.4	2.8
**rP**	76.3	18.9	4.8
**rPG**	80.9	16.9	2.2

Table S2 Surface area and porosity of PBC, rP and rPG samples

Sample	S_{BET} (m^2g^{-1})	S_{mic} (m^2g^{-1})	S_{mic}/S_{BET}	V_{tot} (cm^3g^{-1})	V_{mic} (cm^3g^{-1})	V_{mic}/V_{tot} (%)	Average pore diameter (nm)
**PBC**	336.5	242.8	0.72	0.338	0.111	0.33	4.03
**rP**	264.7	227.8	0.86	0.287	0.104	0.36	4.34
**rPG**	469.9	315.4	0.67	0.667	0.144	0.22	5.68

(a)

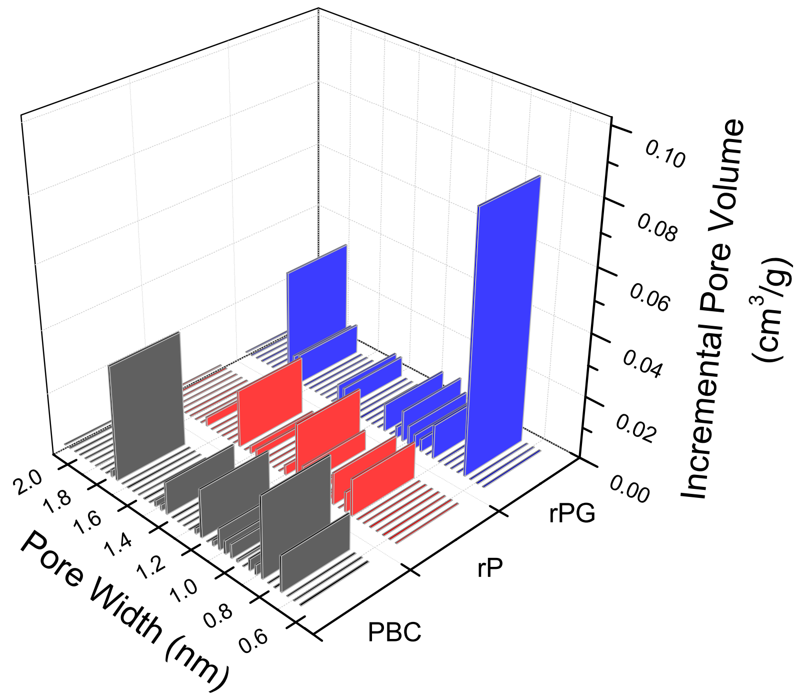

(b)

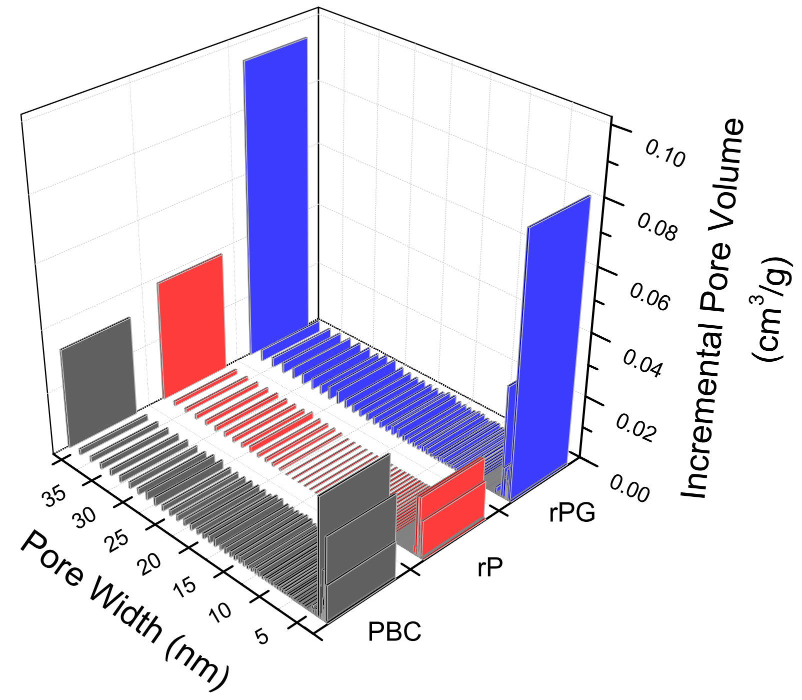

Figure S4 Pore width distribution of PBC,rP,rPG

with the pore width (a) from 0.4 nm to 2 nm (b) from 2 nm to 40 nm

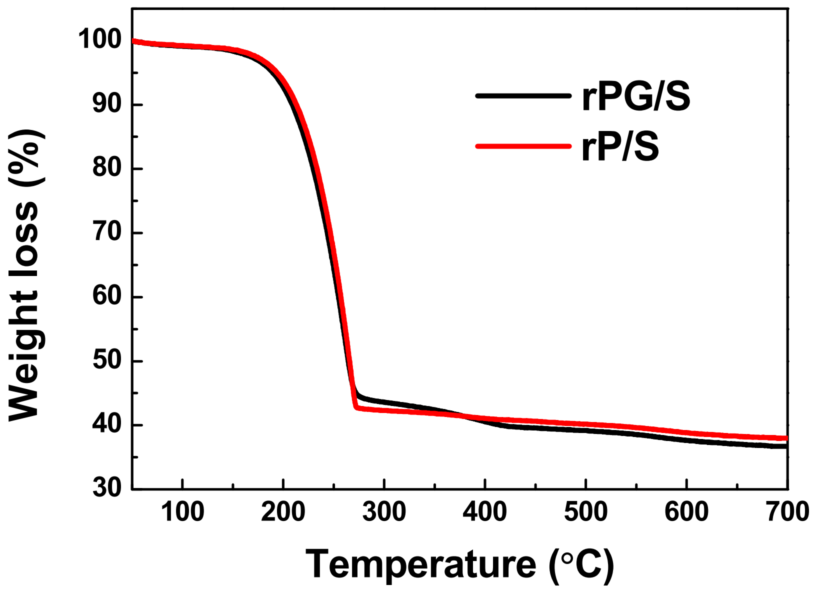

Figure S5. TGA of rP/S and rPG/S under nitrogen atmosphere

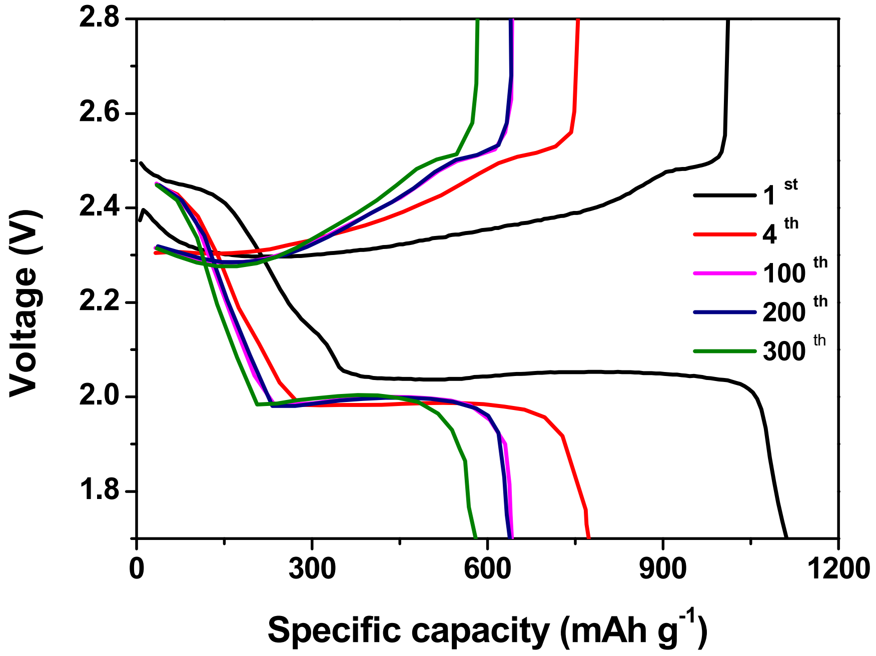

Figure S6. Charge/discharge profiles of rPG/S
